# Myasthenia gravis in an old woman discovered during sedation for diagnostic digestive fibroscopy: Case report

**DOI:** 10.1016/j.amsu.2021.102809

**Published:** 2021-09-04

**Authors:** Habibou rabiou, Idi Djibadje Haboubacar, El Fakhr kaoutar, Liberat Musoni, Hanane Ezzouine, Boubaker Charra

**Affiliations:** a Medical Intensive Care Unit, Ibn Rochd University Hospital, B.P 5696, Casablanca, Morocco; b Faculty of Medicine and Pharmacy, Hassan II University of Casablanca, Morocco

**Keywords:** Myasthenia gravis, Crisis, Old woman, Premedication, Diagnostic management

## Abstract

Introduction and importance: Myasthenia gravis is an autoimmune disease characterized by the destruction of postsynaptic acetylcholine receptors in skeletal striated muscles. It is most common in young women. Myasthenia can be diagnosed by the detection of anti-acetylcholine receptor antibodies. Treatment includes anticholinesterase drugs, thymectomy, and restricting drugs that may aggravate myasthenia. The authors report a rare case of accidental revelation of myasthenia gravis in an elderly woman during sedation for diagnostic gastrointestinal fibroscopy. **Case presentation**: A 85-years-old female patient scheduled for diagnostic gastrointestinal fibroscopy presented signs of myasthenic crisis during the perioperative with severe respiratory failure. The diagnosis of myasthenia was confirmed by bioassay and electromyogram (EMG). Her chest CT scan showed a thymoma. The evolution was favorable as a result of early and appropriate management. **Conclusion:** Myasthenia can occur in perioperative settings outside the usual circumstances. The prognosis depends on early and adapted management.

## Highlights

• Myasthenia gravis is rare in women of advanced age.
• The myasthenic crisis can be inaugural of the disease whatever the age without distinction of sex.
• This case reports a form of discovery of the myasthenic crisis outside the usual circumstance.
• The early diagnosis and the implementation of a medical treatment allow extubating the patient quickly and successfully.

## 1 Introduction

Myasthenia gravis (MG) is an autoimmune disease affecting neuromuscular transmission and resulting in generalized or localized muscle weakness most often due to the presence of autoantibodies directed against acetylcholine receptors in the postsynaptic motor endplate. Myasthenic crisis is a complication of MG characterized by worsening muscle weakness, leading to respiratory failure that requires intubation and mechanical ventilation [1]. The disease classically affects the young female population (sex ratio 2 F/1 M) and is distributed in a bimodal manner, with the first peak in frequency during the second and third decades, affecting mostly women, and a second peak during the sixth and seventh decades, where it affects mostly men [2]. The diagnosis is based essentially on questioning, with confirmation provided by therapeutic tests, biological, electrophysiological, and radiological investigations. In the peri-operative setting, the myasthenic crisis generally occurs in the context of thymectomy surgery [3]. The management of the myasthenic crisis is done in an intensive care unit. The rest of the treatment consists mainly of anticholinesterase drugs, immunoglobulins and thymectomy, and the observation of drug restrictions. The prognosis depends on early and appropriate management [4].

The authors report a rare case of accidental revelation of myasthenia gravis in an elderly woman during sedation for diagnostic gastrointestinal fibroscopy.

## 2 Case report/case presentation

Our work consists of a single case report and was reported by following per under with the SCARE's 2020 criteria [4].

**Fig. 1:**
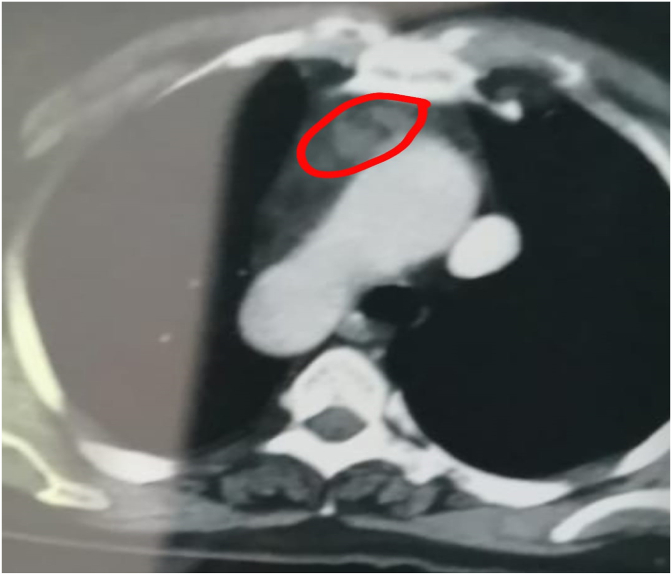
TDM thoracique montrant hypertrophie thymique homogène.

KF, a 85-year-old female patient with unknown medical history, was scheduled for a diagnostic gastrointestinal fibroscopy for a swallowing disorder. She had a pre-procedure pre-anesthetic consultation, which classified her as ASA 1 patient. The pre-fibroscopy chest X-ray was normal and the clinical otorhinolaryngological examination was also unremarkable. The procedure was planned under anesthetic sedation. The patient was pre-medicated with Midazolam (2mg direct intravenous). After induction with titrated propofol (1mg/kg), she presented acute respiratory failure followed by cardiopulmonary arrest (NO FLOW: 0 minute LOW FLOW: 1 minute, Adrenaline: 0 mg. After stabilization, the patient was intubated, ventilated, not sedated, and transferred to the medical intensive care unit. On clinical examination, she was hemodynamically stable with blood pressure at 120/80 mmHg, heart rate (HR) at 95 beats per minute, mean blood pressure at 105 mmHg; respiratory rate at 18 cycles per minute, pulse 97% in the spontaneous mode under 45% Fio2 and a PEEP at 5. The patient had a GCS of 15/15 without motor deficit. She had left ptosis and diplopia. The rest of her somatic examination was characterized by swallowing difficulties. The biological workup was unremarkable with normal gasometry and negative C-reactive protein. Although the criteria for extubation were met on the first day, the first extubation was failed. Chest CT showed homogeneous thymic hypertrophy (Fig. 1) and anti-acetylcholine receptor Ac was positive at 8 nmol/l (norms: 0.4–0.5 nmol/l). The EMG showed the presence of post-synaptic blocks in 3 nerve-muscle pairs in favor of myasthenia. On Anticholinesterase: Pyridostigmine 60 mg/4hours, Immunoglobulin treatment (0.4g/kg/day for 5 days), without corticosteroids after one week, the patient was successfully extubated and transferred to the medical neurology department. By common agreement with the thoracic surgeons, after having obtained the patient's informed and written consent, she was scheduled and operated on for her thymoma, under general anesthesia respecting the contraindications of the anesthetic products. The postoperative evolution was simple and the patient returned home with a neuro medical follow-up note.

## 3 Discussion

Myasthenia gravis (MG) is an autoimmune disease affecting neuromuscular transmission and results in generalized or localized muscle weakness most often caused by autoantibodies to acetylcholine receptors in the postsynaptic motor plate. Myasthenic crisis is a complication of MG characterized by aggravated muscle weakness, which results in respiratory failure requiring intubation and mechanical ventilation [2]. The prevalence of myasthenia is between 5 and 150 cases per million of population, with an incidence of 2.5–20 cases per year per million of population. The disease classically affects the young female population (sex ratio 2 F/1 M) and has a bimodal distribution, with a first peak in frequency during the second and third decades, affecting mainly women, and a second peak during the sixth and seventh decades, where the disease affects mainly men [5].

Contrary to the data of literature, in our case, myasthenia was diagnosed in a woman in her eighth decade.

The vast majority of patients who develop a myasthenic crisis have a precipitating factor such as respiratory infection, emotional stress, microaspirations, a change in medication regime, surgery, or traumatism are among the predisposing factors. However, in 30–40% of cases, no factors are found [2]. Many drugs (Table 1) are known to exacerbate the myasthenic crisis and should be excluded or used with restraint [6]. In our case, the myasthenic crisis was revealing of the disease and probably had several triggers including stress and premedication with midazolam.

**Table 1:** Drugs contraindicated in myasthenia gravis.

Medications formally contraindicated	Medications to be used with precautionary measures
- **Aminoside**	- **Neuroleptics**
- **Colimycin**	- **Benzodiazepines**
- **Polymyxin**	- **Phenothiazines**
- **Cycline injection**	- **Carbamazepine**
- **Quinine - Chloroquine**	
- **Quinidine**	
- **Procai'namide**	
- **Diphenyl-hydantoin**	
- **Trimethadione**	
- **Beta-blockers**	
- **Dantrolene**	
- **d****-PenicilIamine**	
- **All curariforms**	

Myasthenia is rarely discovered accidently. There is some evidence in the literature that myasthenia can be discovered late and accidently in the elderly when using certain forms of antibiotics like tobramycin [7].

In our case myasthenia gravis was discovered accidently during a gastrointestinal fibroscopy diagnosis under sedation following premedication with midazolam.

The use of anaesthetics in perioperative settings is a common situation for decompensation of known myasthenia gravis [3]; however, the discovery of myasthenia gravis as a result of premedication with midazolam during a gastrointestinal fibroscopy in addition to the diagnosis is a rare and unusual situation.

The severity of myasthenia gravis differs from one patient to another and, for the same patient, from one moment to another. Thoracic and diaphragmatic respiratory muscle involvement and severe swallowing disorders characterize the severe forms (20–30% of patients), which can lead to potentially fatal respiratory failure [8].

In our case, the patient presented with a severe form of myasthenia gravis with severe respiratory distress complicated by hypoxia arrest.

The diagnosis of myasthenia gravis is based on interrogation, symptoms, and investigations. The neostigmine (anticholinesterase) test is used to complete the clinical examination. Electromyography (EMG) shows a progressive decrease in the amplitude and duration of the collected potentials during iterative supramaximal stimulations between 2 and 5 Hz. The reduction between the second and fifth potentials must be more than 10% to confirm the diagnosis. The detection of anti-RnACh antibodies by radioimmunoassay, found in 80% of cases, validates the diagnosis [1,2,6,9].

In our case, the symptoms were swallowing disorders, respiratory distress, left diplopia and the anti-acetylcholine receptor antibody assay was positive. The EMG showed the presence of synaptic blocks at the level of three muscle nerve pairs.

The management of a myasthenia gravis crisis requires hospitalization in an intensive care unit for the symptomatic treatment of failures.

The next step in the treatment strategy is always, firstly, to respect the contraindications to medication; secondly, to treat the initiating cause; thirdly, to provide anticholinesterase therapy, immunoglobulins, and possibly plasmapheresis; and fourthly, to proceed with thymectomy [2,4,4,10].

In our case, the patient was managed in an intensive care unit with mechanical ventilation. She was put on anticholinesterase and immunoglobulin. After written consent from the patient, she was then operated on for her thymoma.

The prognosis depends on early and appropriate management [1].

The early and adapted management allowed a successful evolution in our case with the discharging of the patient to her home. The follow-up at three months and then at six months revealed a patient without any symptoms.

## 4 Conclusion

The crisis of myasthenia can be the first episode of the disease regardless of age or sex. Myasthenia can be discovered in the peri-operative period out of the usual situations. The prognosis depends on early and appropriate management.

## Ethical approval

Written informed consent was obtained from the patient for publication of this case report and accompanying images. A copy of the written consent is available for review by the Editor-in-Chief of this journal on request.

## Registration of research studies

1 Name of the registry: researchregistry
2 Unique Identifying number or registration ID: 6921
3 Hyperlink to your specific registration (must be publicly accessible and will be checked):

## Guarantor

Habibou Rabiou

## Provenance and peer review

Not commissioned, externally peer-reviewed.

## Funding

The authors declared that this study has received no financial support.

## Author contribution

Habibou Rabiou: Corresponding author writing the paper.

Idi Djibadje Haboubacar: writing the paper.

Liberat Musoni: writing the paper.

El Fakhr kaoutar1: writing the paper.

Hanane Ezzouine: writing the paper.

Boubaker Charra: Correction of the paper.

## Declaration of competing interest

Authors of this article have no conflict or competing interests. All of the authors approved the final version of the manuscript.
